# Virology laboratory biohazards: infectious MS2 aerosols emitted from common procedures and accidental laboratory scenarios in a controlled chamber study

**DOI:** 10.1128/aem.00408-26

**Published:** 2026-07-21

**Authors:** Ashley R. Ravnholdt, Daniel N. Ackerman, Danielle N. Rivera, Jessica L. Ware, Vicki L. Herrera, Gabriel A. Lucero, Elizabeth A. Klug, Shanna A. Ratnesar-Shumate, Joshua L. Santarpia

**Affiliations:** 1 Department of Pathology, Microbiology and Immunology, University of Nebraska Medical Center 198515, Omaha, Nebraska, USA; 2 Global Center for Health Security, University of Nebraska Medical Center 12284, Omaha, Nebraska, USA; 3 National Strategic Research Institute, Omaha, Nebraska, USA; Centers for Disease Control and Prevention, Atlanta, Georgia, USA

**Keywords:** viral aerosols, virology airborne risks, laboratory aerosol generation, occupational virus exposure

## Abstract

Infectious viral aerosols outside primary containment represent a profound yet undercharacterized occupational biohazard in microbiological environments. While the role of viral aerosols in laboratory-acquired infections is widely acknowledged, quantitative data on the concentration and size distribution of viral aerosols generated during common laboratory workflows remain limited. This study addresses these gaps by using a controlled chamber to characterize the generation of aerosols containing either a physical tracer, sodium fluorescein, or a viral pathogen surrogate, bacteriophage MS2. By integrating aerodynamic particle sizing with cascade impaction and filtered collection, we quantified the mass, number, and infectivity of aerosols emitted during both routine laboratory procedures and simulated accidental releases. These results reveal that every evaluated action generated measurable aerosols spanning multiple particle sizes. Importantly, all MS2-based actions produced infectious aerosols within the respirable size fraction, confirming that routine benchtop pipetting actions yield viral aerosols. These data provide an empirical foundation for evidence-based risk assessments.

**IMPORTANCE:**

Laboratory-acquired viral infections have occurred following routine laboratory procedures in which no distinct accident is recorded, suggesting unrecognized release and inhalation of infectious virus. This research provides a quantitative analysis of these aerosols using a non-pathogenic viral surrogate, MS2, to model viral pathogens. We demonstrate that common laboratory tasks, such as pipetting or handling culture plates, generate respirable infectious aerosols that can bypass standard precautions when outside primary containment. By defining the size and infectious load of these aerosols, this work enables biosafety professionals to transition from qualitative assumptions to data-driven safety protocols, ultimately protecting the personnel responsible for viral pathogen research.

## INTRODUCTION

Viral aerosols within laboratory environments, particularly those outside of containment, represent a significant occupational health hazard. These viral aerosols can potentially lead to laboratory-acquired infections (LAIs) primarily through inhalation exposure. While modern containment measures, such as biosafety cabinets and respiratory protective equipment, are designed to mitigate these risks, LAIs remain an ongoing global challenge. Importantly, laboratory personnel may unknowingly generate infectious aerosols during workflows, where the risk of aerosolization is poorly characterized or underestimated.

LAIs have occurred in the absence of documented protocol breaches or recognized incidents (1). For instance, several documented LAIs involving high-consequence pathogens, including SARS-CoV-1, dengue virus, and Zika virus, followed routine laboratory procedures without a specific aerosol-generating event reported (2–4). Conversely, other cases involving SARS-CoV-1 and Sabiá virus were linked to identifiable spills followed by cleanup activities (5, 6). A review of aerosol-associated LAIs revealed that 101 out of 125 LAI cases were associated with routine procedures rather than discrete accidents (1). This suggests that routine workflows, rather than accidental events, may serve as the primary source of infectious aerosols in laboratory environments.

In microbiology laboratories, both routine tasks (e.g., pipetting) and accidental events (e.g., spills or drops) have reportedly generated bacteria-laden aerosols (1, 7–12). Despite the recognized role of aerosols in virus transmission, existing literature lacks a characterization of viral aerosol generation from laboratory procedures or simulated accidents. Previous studies comparing routine procedures and accidental events have noted that higher-impact events, such as flask drops, release significantly greater concentrations of bacteria-laden aerosols than routine tasks (8). However, these studies often lack a comprehensive characterization of aerosol properties needed for modern microbial risk assessments. Some studies solely reported the count median diameters of viable aerosols (8), which oversimplifies particle size distribution. Relying on a single particle size ignores the complexities of aerosol behavior, including variations in suspension times and site-specific deposition potential within a respiratory tract. Other studies focused on viability-based collection without particle size classification (9–12), limiting the ability to model size-dependent aerosol properties.

Previous laboratory aerosol generation studies are limited to bacterial surrogates, such as *Serratia marcescens* and *Bacillus atrophaeus*, which differ fundamentally from viruses in both physical scale and aerostability (13–15). Virions such as MS2 (~0.03 µm) are orders of magnitude smaller than typical 1 µm bacterial cells (16, 17). These physical differences suggest that viral aerosol generation and subsequent particle size distributions may follow different patterns established in previous bacterial studies (18).

This study aims to address these research gaps by characterizing the concentration, size distribution, and resulting infectivity of viral aerosols generated during representative laboratory procedures and simulated accidents. Representative actions were initially completed with sodium fluorescein (NaFl) as a physical tracer and later with MS2 as a biological surrogate within a controlled testing chamber. By integrating size-resolved aerosol sampling with assays for total and infectious virus, this work provides empirical data to support evidence-based risk assessments. As global research into emerging and high-consequence viruses expands, quantifying viral aerosol generation is needed for promoting safe research practices.

## MATERIALS AND METHODS

### Study design

A controlled experimental study was completed to quantitatively evaluate aerosol generation during representative virology actions, including laboratory procedures and simulated accidents. Actions were completed inside a test chamber using both NaFl and MS2 suspensions. Eleven actions were initially selected based on their relevance to common virology workflows or their implication in LAI reports. Each action was completed with NaFl to screen for aerosol mass generation.

Based on NaFl mass concentrations and action relevance, four actions were chosen for viral aerosol evaluation using MS2. The single-stranded RNA virus MS2 was selected as a conservative surrogate for characterizing mammalian pathogen dynamics due to its established resistance to the mechanical stresses of aerosolization and sampling (18–20). The actions chosen generated the greatest NaFl mass (e.g., plate drop), were considered frequent in virology laboratories (e.g., pipetting), and were likely to be overlooked as significant aerosol sources when completing incident reports (e.g., plate slips). All actions were completed by the authors, who are trained laboratory workers with prior experience in biosafety level 2 operations. Actions were standardized in terms of materials, motion, repetition, and suspension volume.

### Chamber description

**Fig 1:**
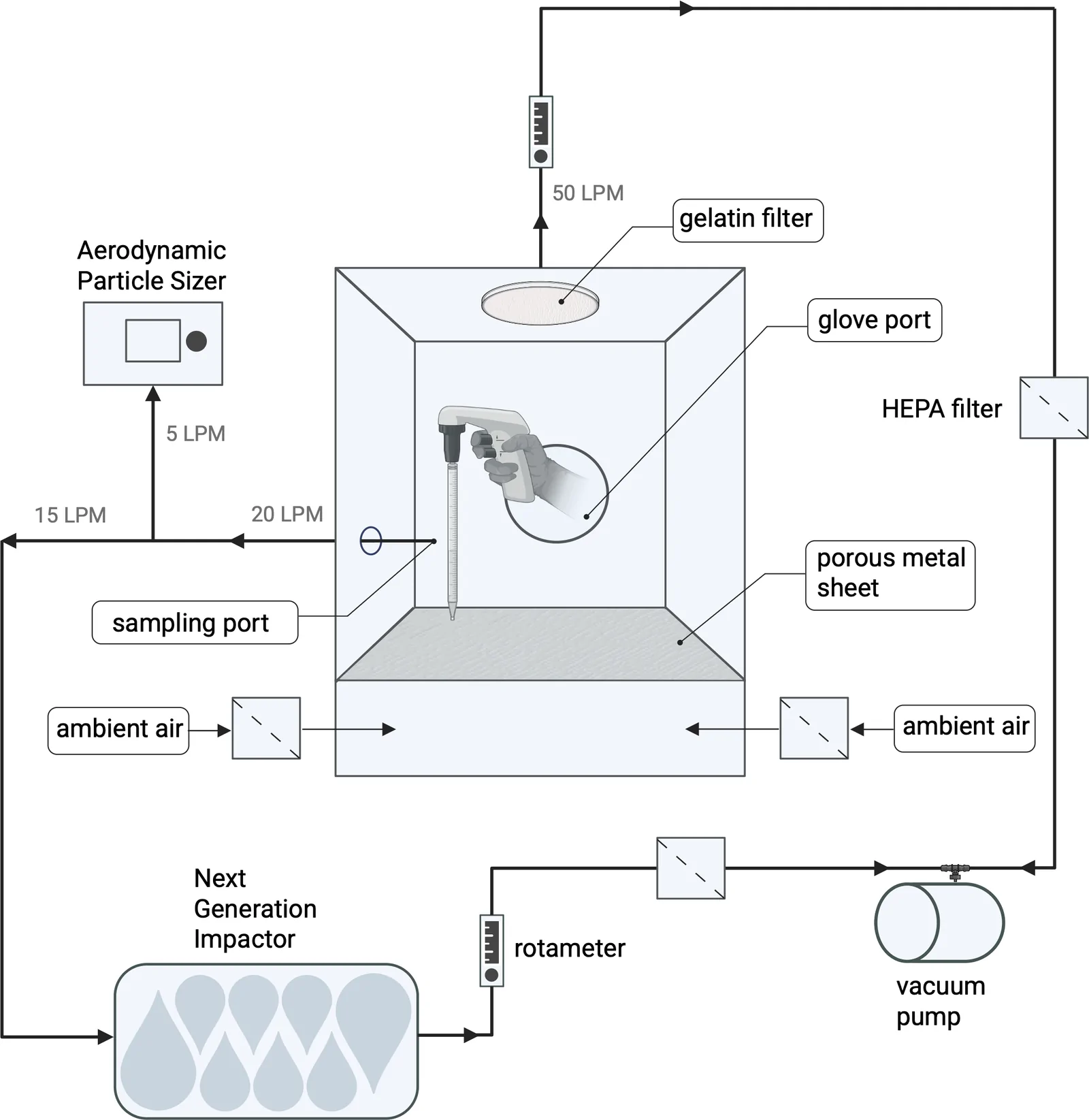
Upwelling aerosol test chamber and integrated sampling system. The chamber consists of a custom-built aluminum enclosure with an internal dimension of 37 cm × 37 cm × 49 cm and a total volume of 67.1 L. The system includes a ceiling-mounted gelatin filter holder, an internal glove port, and a porous metal sheet base to distribute airflow uniformly. A secondary sampling port, constructed from a stainless-steel tube (0.64 cm inner diameter), is installed on the chamber sidewall at a height of 29 cm above the porous base and extends 10 cm into the chamber. HEPA-filtered ambient air enters upward through the porous base and exits via three sampling instruments: a gelatin filter (50 L/min), a cascade impactor (15 L/min), and a particle sizer (5 L/min). Flow rates are controlled by rotameters and driven by a vacuum pump, creating an upward airflow within the chamber.

The test chamber used in this study was a custom-built aluminum enclosure designed to support aerosol generation and sampling (Fig. 1). A 45 cm aluminum extension was added for experiments requiring additional vertical clearance (Table 1).

**TABLE 1:** Experimental action descriptions for sodium fluorescein sampling periods^*a*^

Experimental action	Description	NaFl solution volume (mL)	Repetitions (no. per sampling period)
Invert mixing	Inverted a closed conical tube 10 times, opened briefly, then recapped	5.0	4
Micropipetting	Dispensed solution into microtubes using a P1000 micropipette	0.1 into 0.9	70
Micropipette drop	Released a loaded P1000 micropipette from 38 cm	1.0	3
Micropipetting, rapid	Quickly expelled solution onto a 100 mm dish using a P1000	0.5	30
Plate drop, closed	Released a closed 96-well plate from 84 cm	19.2	1
Plate drop, open	Released an open 96-well plate from 84 cm	19.2	1
Plate drop^*b*^	Released a 6-well plate containing viscous solution from 84 cm	18.0	1
Plate slips	Tilted and released an open 96-well plate from 2 cm	9.6	50
Serological pipetting	Dispensed solution into wells using a 5 mL serological pipette	2.0	10
Serological pipetting^*b*^	Dispensed viscous solution into wells using a 10 mL serological pipette	2.0	6
Vortex mixing	Vortexed a closed conical tube for 10 s, opened briefly, then recapped	5.0	10

^*a*^ This table outlines the procedures and simulated accidents assessed in preliminary NaFl tracer tests. Each action was completed using a defined NaFl volume and repeated within the sampling period to reach the detection limit.

^*b*^ Action was completed using the increased viscosity NaFl solution.

The chamber featured a glove port for internal handling, an access door with a viewing window, multiple sampling ports, and a ceiling-mounted filter holder (Fig. 1). A porous metal sheet (Mott Corporation, USA) was positioned 7 cm above the chamber base to promote vertical airflow, reduce particle settling, and enhance sampling of polydisperse aerosols. Temperature and relative humidity within the chamber were monitored during all experiments using a digital sensor positioned at a chamber ceiling sampling port (DTH3007SD, General Tools, USA). To reduce background aerosol levels, ambient air was drawn through high-efficiency particulate air (HEPA) filters before starting the experiments. Airflow was maintained using a vacuum pump (S55JXTJN8535, Nidec Motor Corporation, USA), with flow rates controlled by rotameters (RMA-25-SSV, Dwyer Instruments, USA). The average vertical velocity in the chamber was 30 cm/s while the sampling instruments were operating (BT-816B, BTMETER, China).

### Aerosol solution preparation

#### NaFl tracer

Sodium fluorescein (NaFl; F5023-25G, Aqua Solutions, USA) was prepared at a 100 µg/mL concentration in filter-sterilized deionized water (SCGP00525, Sigma-Aldrich, Germany). For experiments involving increased viscosity (noted by an asterisk in Table 1), a 1:1 mixture of the NaFl solution and 2.5% microcrystalline cellulose solution (Avicel RC591, DuPont, USA), a common overlay medium used in virology, was combined.

#### MS2 virus model

*Escherichia coli* bacteriophage MS2 (ATCC 15597-B1) was propagated in its host strain, *E. coli* (ATCC 15597). Host colonies were isolated from a streaked agar plate, inoculated into 10 mL of Luria-Bertani (LB) broth (244610, Becton, Dickinson and Company, United States), and incubated overnight in a shaking incubator at 37°C and 180 rpm. The resulting overnight culture was diluted 1:100 in 5 mL aliquots of fresh LB broth and grown to mid-log phase (OD_600_ of 0.45 to 0.55), with growth monitored via spectrophotometry at 600 nm (6131, Eppendorf, Germany). To initiate viral infection, a 100 µL aliquot of MS2 stock was inoculated into each 5 mL mid-log phase host culture and incubated overnight in a shaking incubator at 37°C and 180 rpm. Following propagation, the lysate was centrifuged at 4,000 × *g* for 10 min to pellet cellular debris. The resulting supernatant was passed through a 0.22 µm polyethersulfone membrane (SCGP00525, Sigma-Aldrich, Germany) to yield the filter-sterilized MS2 viral suspension. Viral stocks were stored at 4°C and quantified by plaque assays on the day of each experiment.

### Aerosol generation and run procedures

Aerosol generation in this study was contingent on whether the experimental actions produced aerosols. However, to ensure consistency and assess sampling efficiency across experimental runs, a standardized aerosolization method was applied. For this purpose, each aerosol solution was aerosolized within the test chamber using a jet nebulizer (HUD1882, Teleflex, USA), powered by a portable compressor (6910, Vega Technologies, Taiwan). To represent where the experimental actions were completed within the chamber, the nebulizer was centrally positioned atop the porous metal base inside the chamber.

During each sampling period, the following equipment was activated simultaneously: the particle sizer, chamber flow vacuum, and temperature/relative humidity sensor. Several actions required repeated actions within a single sampling period to generate detectable NaFl mass concentrations. The number of repetitions varied by experimental action and is detailed in the “Repetitions” column of Table 1. Each sampling period was completed in triplicate.

A subset of actions from the NaFl runs were repeated using MS2 to evaluate viral aerosol characteristics. Table 2 summarizes the experimental parameters, including the name and description of each action, the volume of viral suspension used, and the number of repetitions performed during each sampling period. Sampling periods were completed in triplicate for each experimental action.

**TABLE 2:** Experimental action descriptions for MS2 sampling periods^*a*^

Experimental action	Description	Viral solution volume (mL)	Virus stock, initial concentration (PFU/mL)	Repetition (no. per sampling period)
Micropipetting	Transferred solution across 96-well plate rows using a 12-channel micropipette	0.1	3.20 × 10^10^	96^*b*^
Serological pipetting	Aspirated and dispensed with a 5 mL pipet in an open conical tube	2.0	7.25 × 10^11^	50
Plate slips	Tilted and released an open 96-well plate from 2 cm	9.6	3.27 × 10^11^	300
Open plate drop	Released an open 96-well plate from 38 cm	19.2	2.57 × 10^13^	1

^*a*^ These procedures and simulated accidents were selected for viral aerosol testing based on NaFl results.

^*b*^ A multichannel micropipette with 12 tips was used to transfer 0.1 mL per tip across eight rows, totaling 96 transfers per sampling period.

Between each sampling period, the chamber interior was sprayed with a commercial decontamination solution (10328011, Invitrogen by Thermo Fisher Scientific, Singapore), followed by a 70% alcohol rinse. The chamber was then actively vented for at least 30 min until particle counts returned to baseline background levels. To verify that the air within the enclosure was clear of residual airborne contaminants, negative control samples were collected from the NGI after the chamber was vented.

### Aerosol collection and measurement

Preliminary NaFl studies involved simultaneous aerosol sampling at two locations within the chamber. The primary collection site was at the chamber ceiling, where gelatin filters (17528-80-ACD, Sartorius, Germany) sampled the main upwelling airflow at 50 liters per minute (L/min). The filter holder was installed inside the chamber ceiling to maximize collection efficiency and reduce particle loss associated with external tubing (Fig. 1).

A secondary sampling location was positioned on the chamber sidewall, where a sampling port extended into the chamber to direct aerosols to a cascade impactor, a Next Generation Impactor (NGI; 0170-01-1000, TSI Inc., USA). Aerosol size distributions were characterized using an NGI at a 15 L/min flow rate. The NGI separated aerosols across eight stages by aerodynamic diameter (Table 3). Each stage is defined by its respective cut-off diameter (d_50_), representing the median aerodynamic diameter at which 50% of particles are collected.

**TABLE 3:** Particle sizes collected on the Next Generation Impactor (NGI) stages at 15 L/min^*a*^

Stage number	Size range of collected particles (µm)
1	>14.10
2	8.61–14.10
3	5.39–8.61
4	3.30–5.39
5	2.08–3.30
6	1.36–2.08
7	0.98–1.36
Micro-orifice collector	0–0.98

^*a*^ The cut-off diameters for the NGI at 15 L/min were previously derived (21). The effective collection range for each stage spans from its d_50_ to the d_50_ of the preceding stage.

Later, an Aerodynamic Particle Sizer (APS; 3321, TSI Inc., USA) operating at 5 L/min was incorporated to obtain high-resolution aerosol size distributions, as the APS provides narrower bin widths than the NGI. Both the APS and NGI were connected to a single shared sampling port (Fig. 1), yielding a combined flow rate of 20 L/min at the port. The gelatin filter remained positioned at the chamber ceiling, collecting aerosol at 50 L/min. This optimized sampling configuration was applied to the seven NaFl experimental actions presented in Fig. 4 and all four MS2 actions. To preserve viral viability during MS2 experiments, each NGI stage was coated with 1 mL of sodium magnesium (SM) buffer.

### Sample extraction

#### NaFl samples

Gelatin filters were dissolved in 5 mL of diluent (filter-sterilized deionized water) and placed in a shaking incubator at 37°C for approximately 5 min to liquefy the gelatin. For NGI samples, 10 mL of diluent was added to the first and final stages, and 5 mL to each of the six intermediate stages. Each stage was gently rocked to ensure uniform coating and sample recovery. Aliquots were collected from all samples for downstream analysis.

#### MS2 samples

All MS2 samples were processed in SM buffer. Gelatin filters were dissolved in 10 mL of SM buffer under the same incubation conditions (37°C, shaking, ~5 min). For NGI stages, 9 mL of SM buffer was added to each NGI stage, gently rocked for even coating, and transferred to a vial. Each sample was split for downstream quantification via plaque assay and RNA extraction before quantitative reverse transcription polymerase chain reaction (RT-qPCR). RNA extractions were completed the same day as collection (955134, Qiagen, Netherlands).

### Sample analysis

To quantify NaFl, samples were measured in triplicate using a Qubit 4 Fluorometer (Q33226, Invitrogen by Thermo Fisher Scientific, Singapore) with an excitation wavelength of 470 nm, capturing raw fluorescence units (RFU) emission within the 510–580 nm range. RFU readings were converted to NaFl mass using a linear regression equation derived from a standard curve created from a series of 2-fold dilutions of known NaFl concentrations. For each sample type (gelatin filter and NGI stages), blank controls were read, and the average RFU was subtracted from sample measurements to calculate the recovered NaFl mass from each sample:


$$NaFl\;recovered=\;\left(\frac{{\overset{-}{X}}_{RFU}-\;B_{RFU}}{m}\right)\times V_{R}$$

where ${\overset{-}{X}}_{RFU}$ represents the average RFU from the sample, *B*_RFU_ denotes the average RFU from blank controls, and m is the slope from the standard curve (*m* = 45,867, *R*^2^ = 0.9996). The recovery volume (*V_R_*) accounted for the total sample volume collected.

Infectious MS2 concentrations were quantified using double-layer plaque assays. Recovered samples were mixed with *E. coli* host cultures (grown to an OD_600_ of 0.4–0.7) and incubated for 12 min at room temperature with continuous shaking at 180 rpm to promote viral adsorption. Following incubation, 4 mL of 0.7% top agar (BP1423-500, Fisher Bioreagents by Thermo Fisher Scientific, United States) was added to the mixture, which was immediately overlaid onto 1.5% agar LB plates. Plates were incubated overnight at 37°C, and visible plaque-forming units (PFU) were counted the following day.

Infectious viral aerosol concentrations, reported as PFU per liter of air (PFU/L air), were calculated using the following equation:


$$\frac{PFU}{L\;air}=\left(\frac{C\times V_{T}}{\left(LPM\times min\right)}\right)$$

where *C* is the average viral titer in PFU/mL, *V*_*T*_ is the total recovered sample volume, LPM is the sampling flow rate, and min is the total sampling duration.

Viral RNA was quantified as a measure of total MS2 virus using RT-qPCR. Thermal cycling conditions included an initial 10-minute hold at 55°C, followed by a 4-minute hold at 94°C and 45 cycles of 94°C for 15 s and 58°C for 30 s. Each reaction contained 6.1 µL nuclease-free water, 12.5 µL of 2X Invitrogen RT-PCR Reaction Mix, 0.4 µL of 50 mM MgSO_4_, 0.5 µL of primer/probe mix consisting of a forward primer (5′-GTCGCGGTAATTGGCGC-3′), a reverse primer (5′-GGCCACGTGTTTTGATCGA-3′), and a probe (5′-AGGCGCTCCGCTACCTTGCCCT-3′), 0.5 µL of Superscript III RT/Platinum Taq Mix, and 5 µL of extracted RNA samples.

Cycle threshold (Ct) values were converted to equivalent viral titers (ePFU/mL) using an exponential equation derived from a standard curve generated using a 9-log, 10-fold serial dilution series of plaque-quantified MS2. The resulting standard curve equation, $\frac{ePFU}{mL}=1\times 10^{14}\cdot e^{-0.75\cdot Ct}$ (*R*^2^ = 0.9998), was used to estimate viral concentrations based on measured Ct values. Equivalent titers were then converted to viral aerosol concentrations, reported as viral equivalent PFU per liter of air (ePFU/L air), using the following equation:


$$\frac{ePFU}{L\;air}=\left(\frac{C\times V_{T}}{\left(LPM\times min\right)}\right)$$

where *C* is the average equivalent viral titer in ePFU/mL, *V*_*T*_ is the total recovered sample volume, LPM is the sampler flow rate, and min is the sampling duration.

Negative controls included nuclease-free water, SM buffer, blank gelatin filters, and clean NGI stages. A positive control, prepared from standard curve dilutions, was included on each PCR plate to ensure consistency across sample quantification.

To assess the average difference between total and infectious virus concentrations across all air samples, log differences were calculated as the mean ± standard deviation of ΔLog_10_[(ePFU/L Air) − (PFU/L Air)]. Viral particle size distributions were evaluated by plotting viral aerosol concentrations from RT-qPCR and plaque assays against the aerodynamic cut-off diameters associated with each NGI stage.

Statistical comparisons for gelatin filter and NGI results were completed using two-way analysis of variance (ANOVA), followed by Tukey’s multiple comparison tests, evaluating differences across group means and across sampling stages.

### Virus exposure risk assessment

Virus exposure risk was estimated using aerosol concentrations measured at the gelatin filter, expressed as either PFU/L (infectious virus) or ePFU/L (total virus). The estimated inhaled dose was determined by multiplying the measured concentration by an average adult inhalation rate (9.5 L/min [22]) and an exposure duration of 10 min. This calculation assumes a uniform aerosol distribution within the chamber and does not account for laboratory ventilation dynamics or environmental factors (e.g., temperature and humidity).

## RESULTS

### NaFl screening results

**Fig 2:**
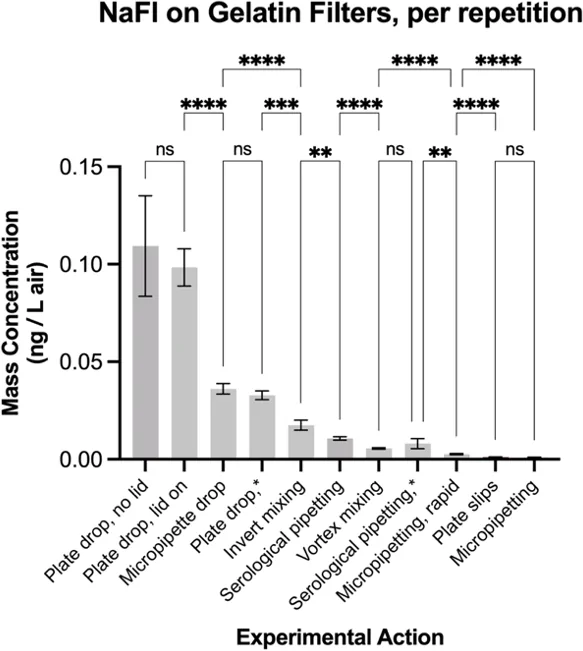
NaFl mass concentration per repetition on gelatin filters. The 11 experimental actions tested with NaFl are plotted based on the mass concentration of NaFl on the gelatin filters per repetition. Results of one-way ANOVA with a multiple comparisons test. Asterisks (*) indicate the experimental actions were statistically significant in difference from another, while groups that were not significant are labeled ns. Open plate drops are noted as no lid, while closed plate drops are noted as lid on.

All 11 laboratory procedures and simulated accidents evaluated during the NaFl screening phase generated measurable aerosol mass concentrations on the gelatin filters (Fig. 2). Nine of these actions also produced measurable NaFl across all eight NGI stages, indicating broad particle size distributions.

When ranked by statistically significant differences in NaFl mass concentration per repetition, plate drops (open and closed) produced the highest concentrations, followed by micropipette drops, viscous plate drops, and invert mixing (Fig. 2). Serological pipetting was next, followed by vortex mixing and viscous serological pipetting, with rapid micropipetting and plate slips producing lower concentrations (Fig. 2). Standard micropipetting generated the lowest NaFl mass per individual repetition (Fig. 2).

**Fig 3:**
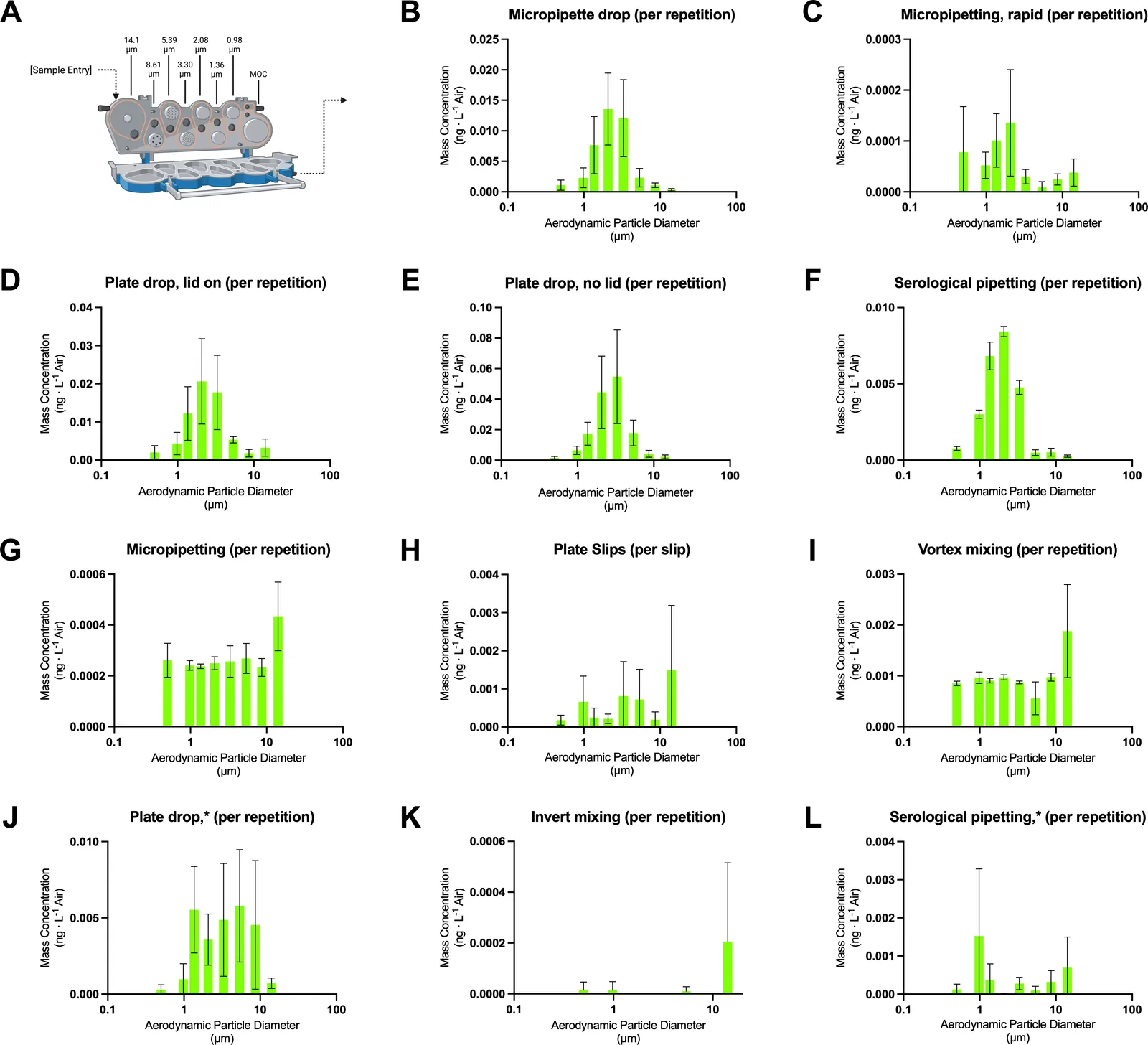
NaFl mass distribution across NGI stages during select laboratory activities. Individual bar plots displaying NaFl mass concentrations per repetition across NGI stages for each experimental action (detailed in Table 1). (**A**) NGI with associated stage cutoff sizes. (**B–L**) Each plot reflects NaFl mass concentration mean ± 95% confidence interval at each stage per repetition of the action. Asterisks (*) indicate actions using a more viscous NaFl solution. Open plate drops are noted as no lid, while closed plate drops are noted as lid on.

Most actions exhibited distinctive particle size distribution profiles, with NaFl mass found on more than one stage for all actions (Fig. 3).

Micropipette drops, rapid micropipetting, and closed plate drops exhibited relatively unimodal mass distributions, with peaks near the 2.08 µm NGI stage (Fig. 3B through D). Open plate drops and serological pipetting also followed unimodal profiles but peaked at 3.30 µm (Fig. 3E and F). Notably, the highest NaFl concentration observed across all NGI samples occurred at 3.30 µm during the open plate drop action (Fig. 3E). Standard micropipetting, plate slips, and vortex mixing displayed relatively uniform mass distributions across all NGI stages, with slight peaks observed at 14.1 µm (Fig. 3G through I), suggesting consistent generation of both fine and large particles. Viscous plate drops produced a broad mass distribution across the intermediate stages (2–7), with reduced mass at the smallest and largest particle sizes (Fig. 3J). Invert mixing and viscous serological pipetting demonstrated bimodal profiles, with peaks at both 0.98 and 14.1 µm (Fig. 3K and L), indicating simultaneous generation of submicron and relatively larger aerosol particles.

**Fig 4:**
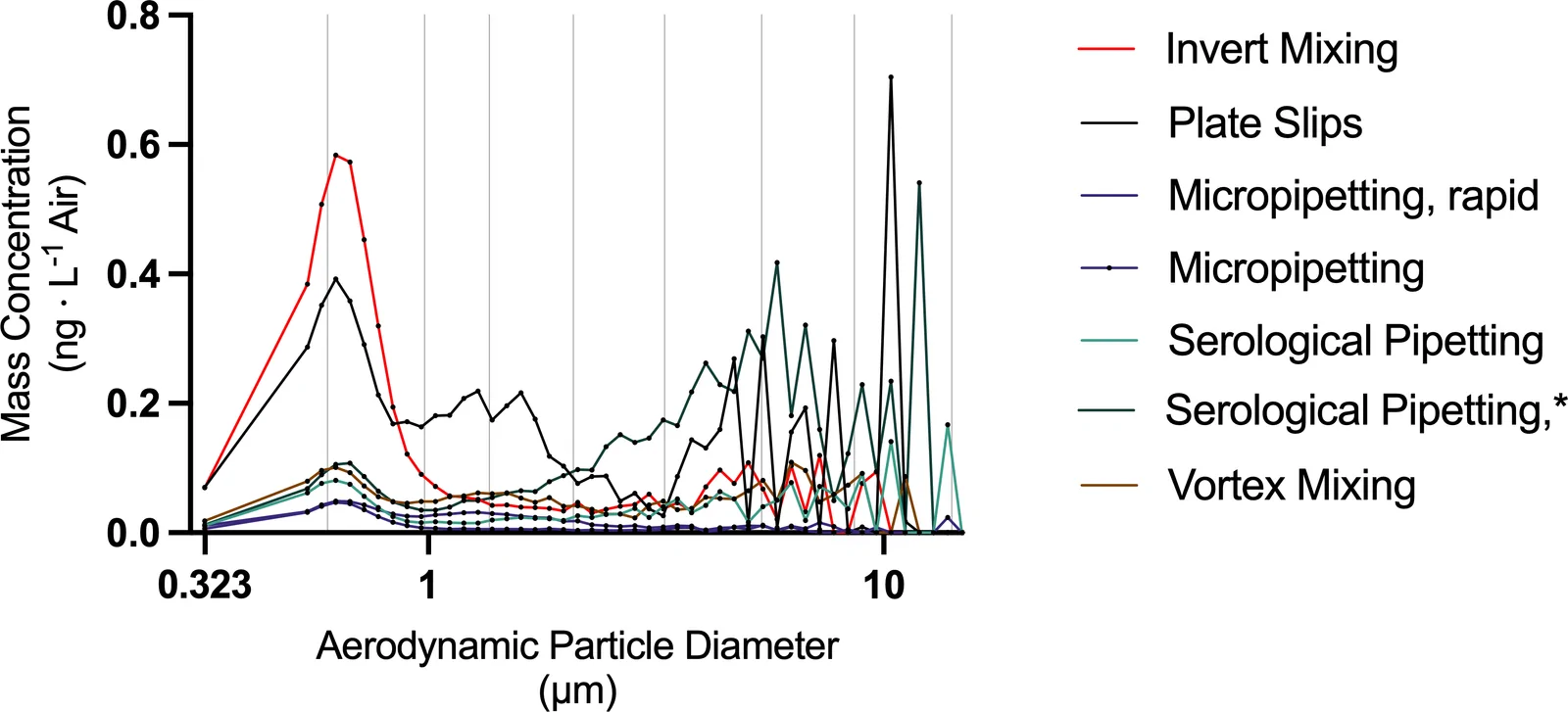
NaFl mass detected across APS channels during select laboratory activities. Mass distribution across aerodynamic particle diameters for each experimental action, measured using an APS. Each colored line corresponds to an action, as indicated in the legend. Data represent the average of three replicates per action. Vertical lines indicate the cut-off diameters for the eight NGI stages, included to compare between APS and NGI mass recovery.

The APS measurements complemented the NGI particle size distributions with broad particle sizes for each action. However, a fine particle mass peak near 0.6 µm was observed for all actions (Fig. 4).

Additionally, qualitative surface examination under a UV light revealed localized droplets on the chamber surfaces, specifically noted after plate drops and pipette drops. This indicated the simultaneous generation of larger droplets that fell out of suspension before reaching the sampling instruments.

### MS2 viral surrogate results

Based on the NaFl results, four representative actions were selected for further assessment using MS2 to assess infectious and total virus aerosolized. These actions included micropipetting, serological pipetting, plate drops, and plate slips, which were chosen for their relevance to common laboratory practices and known implications in LAIs. The RNA sampling efficiency following nebulization was 72.0% ± 8.5% (mean ± SEM, *n* = 3). Across all experimental actions, total viral aerosol concentrations (measured as ePFU by RT-qPCR) exceeded infectious viral aerosol concentrations (PFU by plaque assay). Across all samples recovered from the NGI, this difference was 1.42 ± 0.36 log_10_ higher in total than infectious virus. Since the RT-qPCR standard curve was constructed using the same plaque-quantified stock suspensions used for experimental runs, any baseline exogenous RNA present in the initial suspension was inherently normalized. Consequently, this ensures that downstream differences between total and infectious titers reflect the effect of mechanical forces from aerosolization and sampling collection rather than variance in the starting material. Although this pattern was consistent, the difference between concentrations did not reach statistical significance. This result suggests the presence of viral RNA from non-infectious or fragmented viral particles that are detectable by PCR but not culturable by plaque assay.

Few statistically significant differences emerged when comparing viral aerosol concentrations on gelatin filters between experimental actions. Notably, total virus aerosol concentrations (ePFU/L air) recovered from plate drops were significantly higher than those from plate slips, serological pipetting, and micropipetting (*P* < 0.05 for all comparisons).

**Fig 5:**
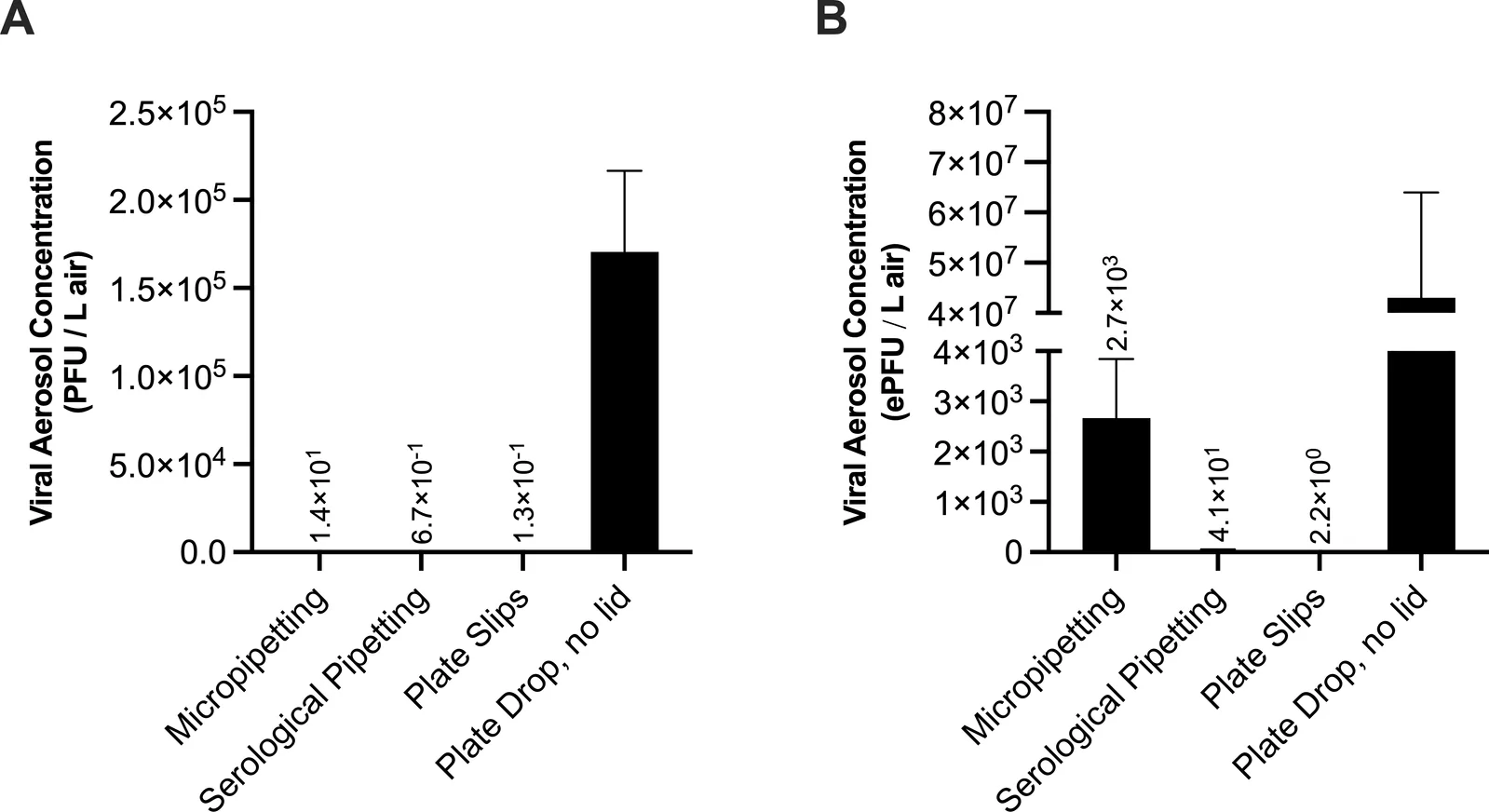
MS2 virus concentration per repetition on gelatin filters. Mean viral aerosol concentrations are presented as active infectious units (PFU/L air) (**A**) and genomic equivalents (ePFU/L air) (**B**) for each experimental action with MS2. Error bars represent the standard error of the mean across independent biological replicates (*n* = 3). Statistical variance was evaluated via a two-way ANOVA with a pos-hoc multiple comparisons test.

Additionally, within plate drop experiments, viral RNA aerosol concentrations (ePFU/L air) significantly exceeded infectious aerosol concentrations (PFU/L air; Fig. 5).

While plate drops produced the highest infectious aerosol concentrations, followed by micropipetting, serological pipetting, and plate slips, these differences among actions were not statistically significant. Notably, the plate drop action consistently produced the highest aerosol concentrations measured at the gelatin filter across both NaFl and MS2 experiments.

Size-resolved physical and biological aerosol characteristics varied significantly across the evaluated experimental workflows, yet revealed a shared underlying particle size distribution (Table 4). The APS detected aerosol generation across all channels for every MS2 action, featuring a consistent concentration peak near 0.6 µm that matched preliminary NaFl tracer runs and pointed to a mechanical aerosolization mechanism.

**TABLE 4:** Aerosol size distribution peaks and total vs. infectious viral concentrations across experimental actions^*a*^

Experimental action	Micropipetting	Serological pipetting	Plate slips	Plate drops
APS peak number diameter (µm)	1.5	0.6	4.7	0.6
APS peak mass diameter (µm)	6.3	4.4	8.6	5.1
NGI RNA peak (d_50_, µm)	2.08, 14.1	<0.98, 2.08	3.30, 5.39, 8.61	5.39
NGI infectious peak (d_50_, µm)	3.30, 5.39	2.08, 8.61	2.08, 3.30, 5.39	5.39
Gelatin filter RNA (ePFU/L air)	2.7 × 10^3^	4.1 × 10^1^	2.2 × 10^0^	4.3 × 10^7^
Gelatin filter infectious (PFU/L air)	1.4 × 10^1^	6.7 × 10^−1^	1.3 × 10^−1^	1.7 × 10^5^

^*a*^ Peak particle diameters for physical and biological aerosols are presented across four experimental actions with MS2. Continuous particle size distributions were characterized via an Aerodynamic Particle Sizer (APS) to identify peak concentrations by particle count (dN/dLogDp) and mass (dM/dLogDp). Size-resolved viral distributions were evaluated using a Next Generation Impactor (NGI) to identify peak concentrations by aerodynamic cutoff diameter (d_50_) for both viral RNA and infectious units. Volumetric RNA concentrations (ePFU/L air) and infectious concentrations (PFU/L air) collected on gelatin filters represent the mean concentrations across independent biological replicates (*n* = 3).

**Fig 6:**
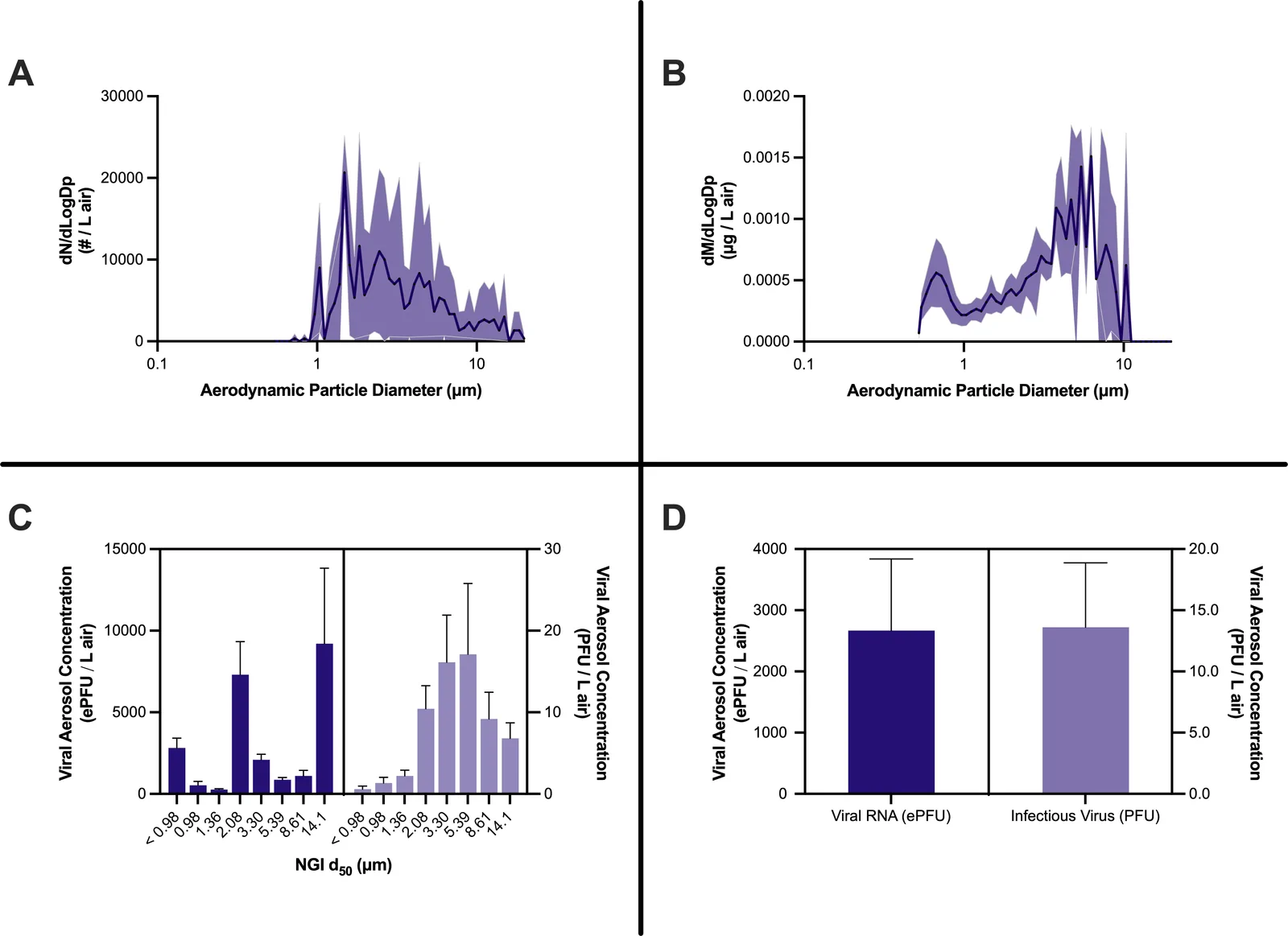
Physical and biological MS2 aerosol characteristics generated during micropipetting. Continuous size distributions are resolved by aerodynamic diameter via APS, reporting mean particle number concentration (dN/dLogDp) (**A**) and mean normalized mass concentration (dM/dLogDp) (**B**). Shaded regions in panels A and B denote the standard deviation at each diameter. (**C**) Size-fractionated viral aerosol concentrations resolved across NGI stages. (**D**) Mean viral aerosol concentrations captured on the gelatin filters. For panels C and D, the left plots present total viral RNA genomic equivalents (ePFU/L air), and the right plots present infectious concentrations (PFU/L air); error bars represent the standard error of the mean.

**Fig 7:**
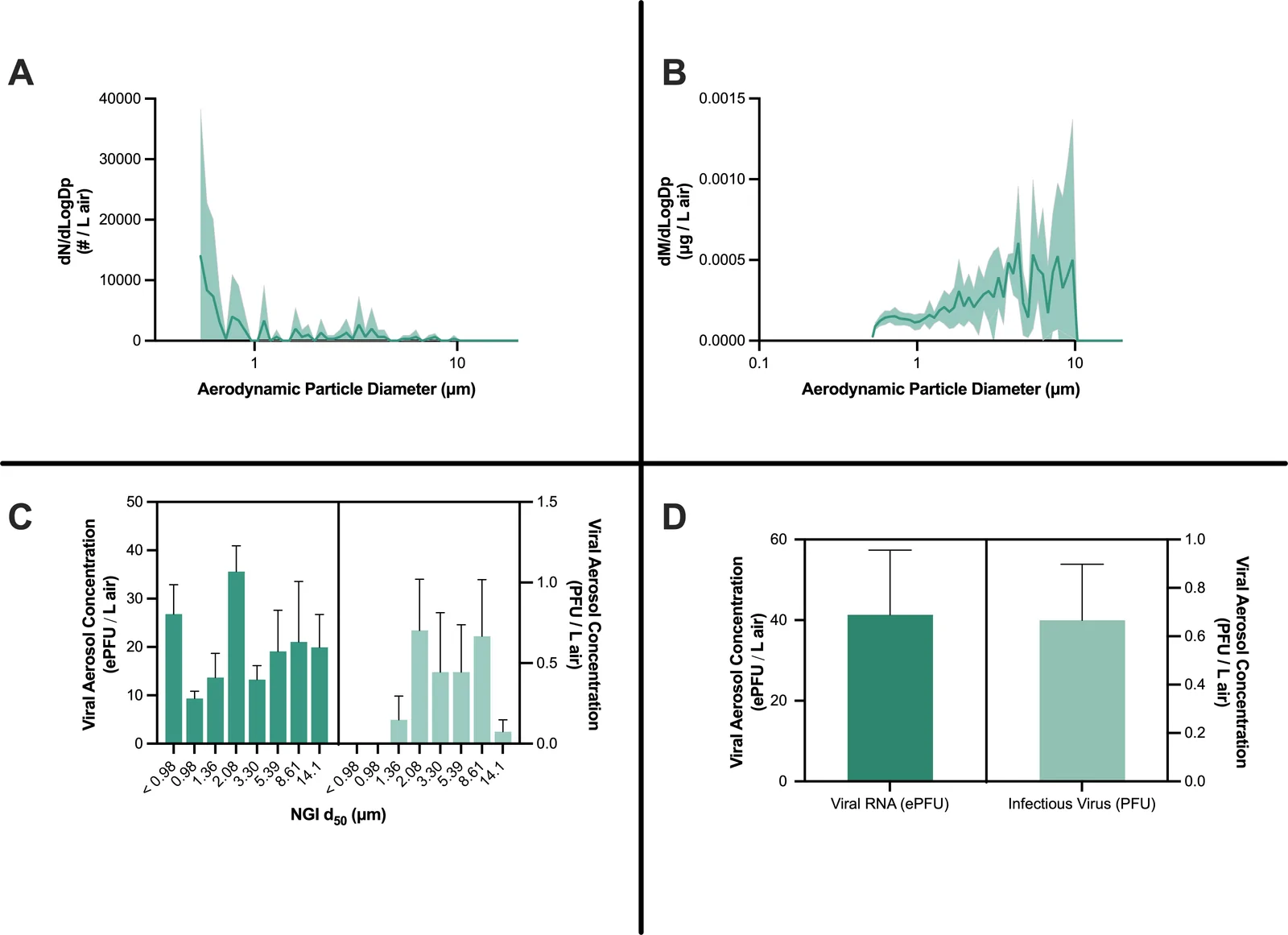
Physical and biological MS2 aerosol characteristics generated during serological pipetting. Continuous size distributions are resolved by aerodynamic diameter via APS, reporting mean particle number concentration (dN/dLogDp) (**A**) and mean normalized mass concentration (dM/dLogDp) (**B**). Shaded regions in panels A and B denote the standard deviation at each diameter. (**C**) Size-fractionated viral aerosol concentrations resolved across NGI stages. (**D**) Mean viral aerosol concentrations captured on the gelatin filters. For panels C and D, the left plots present total viral RNA genomic equivalents (ePFU/L air) and the right plots present infectious concentrations (PFU/L air); error bars represent the standard error of the mean.

**Fig 8:**
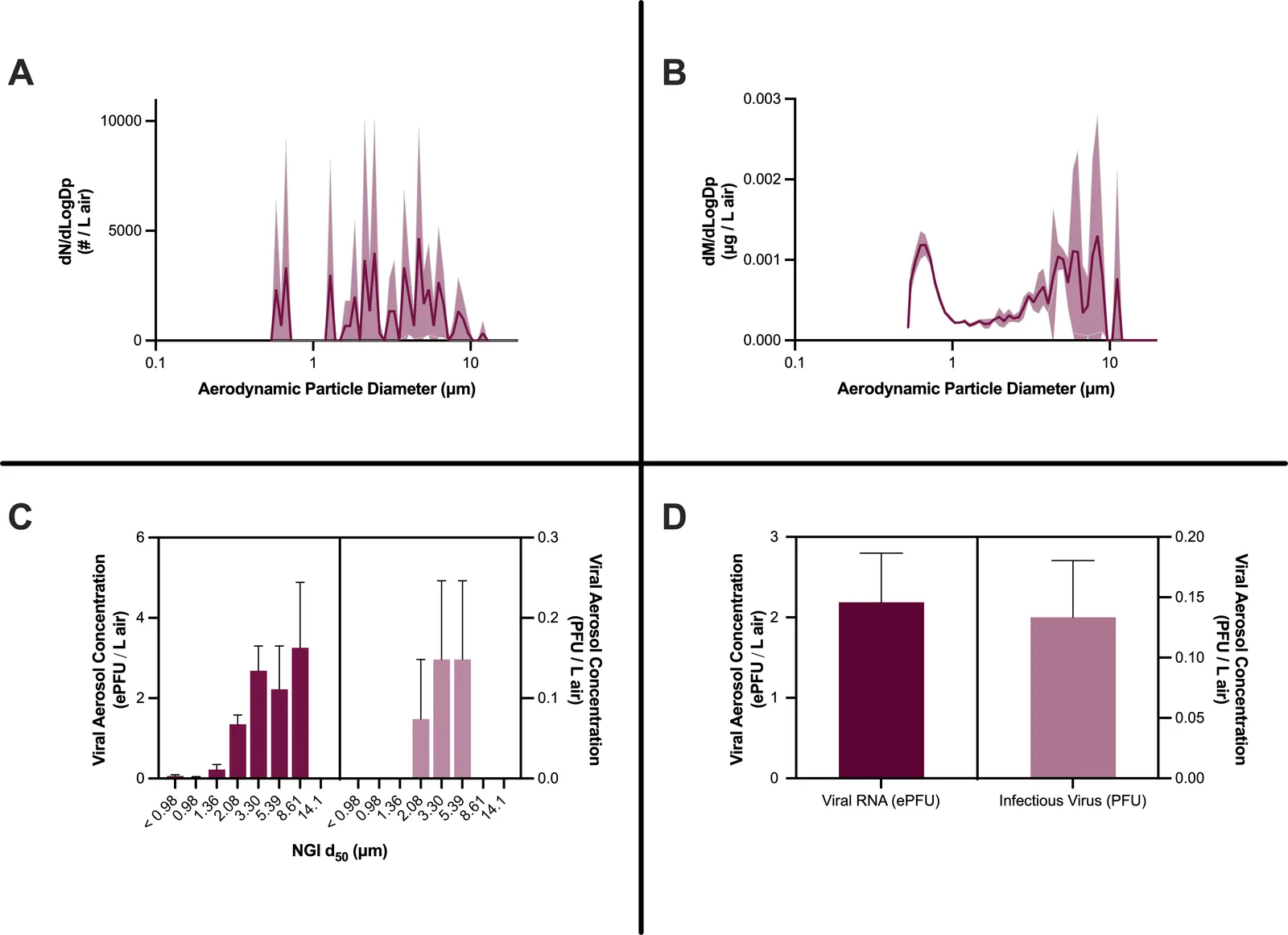
Physical and biological MS2 aerosol characteristics generated during plate slips. Continuous size distributions are resolved by aerodynamic diameter via APS, reporting mean particle number concentration (dN/dLogDp) (**A**) and mean normalized mass concentration (dM/dLogDp) (**B**). Shaded regions in panels A and B denote the standard deviation at each diameter. (**C**) Size-fractionated viral aerosol concentrations resolved across NGI stages. (**D**) Mean viral aerosol concentrations captured on the gelatin filters. For panels C and D, the left plots present total viral RNA genomic equivalents (ePFU/L air) and the right plots present infectious concentrations (PFU/L air); error bars represent the standard error of the mean.

**Fig 9:**
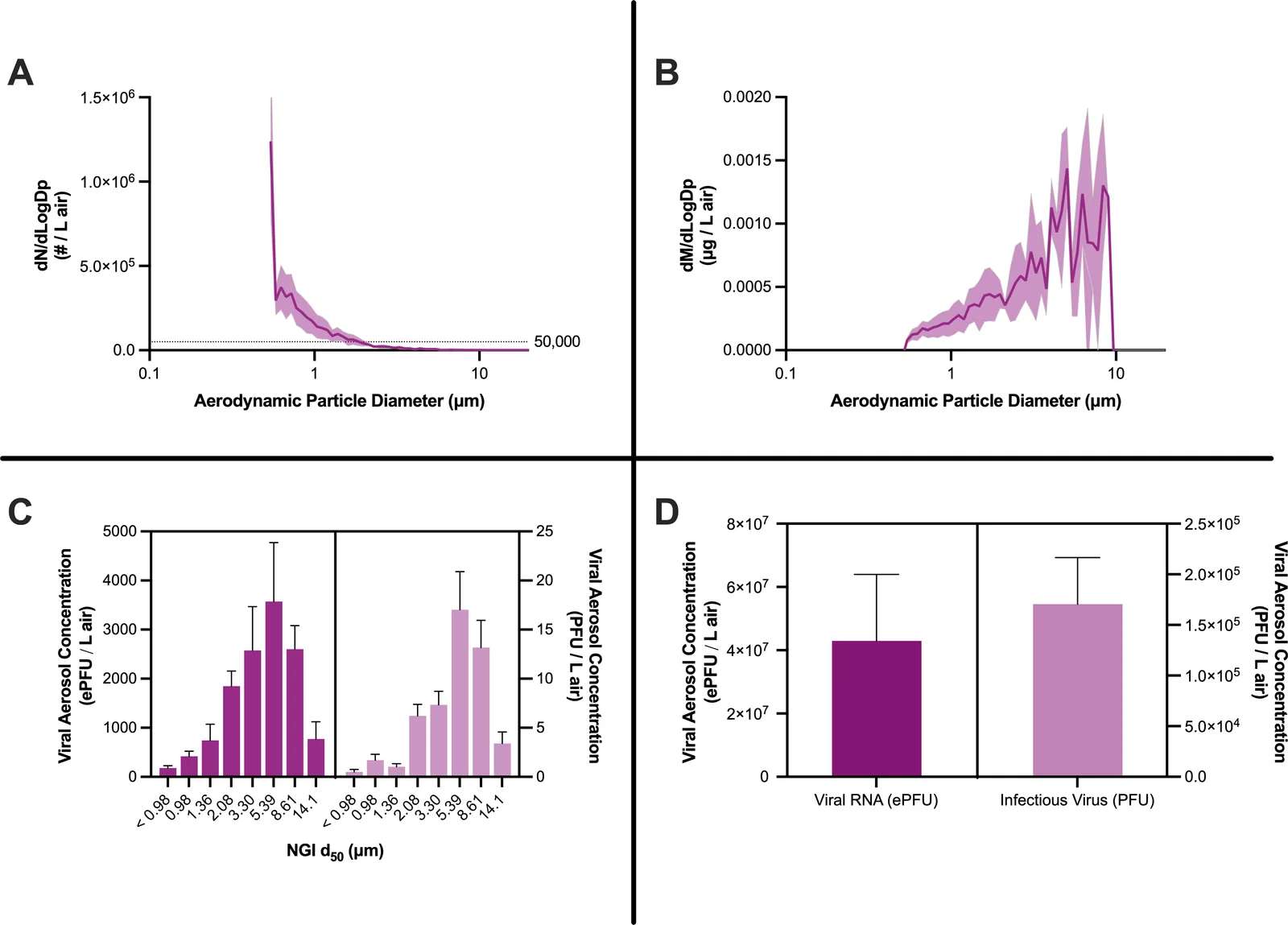
Physical and biological MS2 aerosol characteristics generated during open plate drops. Continuous size distributions are resolved by aerodynamic diameter via APS, reporting mean particle number concentration (dN/dLogDp) (**A**) and mean normalized mass concentration (dM/dLogDp) (**B**). Shaded regions in panels A and B denote the standard deviation at each diameter. (**C**) Size-fractionated viral aerosol concentrations resolved across NGI stages. (**D**) Mean viral aerosol concentrations captured on the gelatin filters. For panels C and D, the left plots present total viral RNA genomic equivalents (ePFU/L air) and the right plots present infectious concentrations (PFU/L air); error bars represent the standard error of the mean.

Biologically, total viral RNA (ePFU/L air) demonstrated broad size distributions, recovering across all eight NGI stages for micropipetting, serological pipetting, and plate drops (Fig. 6C, 7C and 9C), and across seven stages for plate slips (Fig. 8C).

However, infectious virus recovery was constrained by particle diameter. Notably, during serological pipetting, no viable virus was isolated from the submicron fractions (<0.98 µm and 0.98 µm) despite high RNA recovery (Fig. 7C). Similarly, plate slips did not yield infectious virus at the 8.61 µm stage despite high RNA concentrations (Fig. 8C). Conversely, micropipetting and accidental plate drops yielded persistent viable virus across every stage of the NGI, demonstrating that these experimental actions efficiently produce infectious aerosols across both respirable and inhalable size ranges (Fig. 6C and 9C). Bulk collection on the gelatin filters established the boundaries of exposure, with simulated plate drops generating the highest infectious burden and plate slips yielding the lowest (Table 4).

Virus exposure estimates for occupational risk assessment were derived from viral concentrations measured at the gelatin filter. Although the NGI offers size-resolved viral data, the gelatin filter sampled a significantly higher volumetric flow (50 L/min compared to 15 L/min for the NGI), thereby capturing a more comprehensive representation of the total viral aerosol generated during each action. These exposure estimates, reflecting inadvertent aerosol generation during laboratory procedures and accidents, are presented in Table 5.

**TABLE 5:** Estimated inhalation exposure to infectious and total virus from laboratory procedures and accidents^*a*^

Experimental action	Estimated exposure to infectious virus (PFU)	Estimated exposure to viral particles (ePFU)
Micropipetting	1.24 × 10^3^	2.57 × 10^5^
Serological pipetting	6.37 × 10^1^	3.90 × 10^3^
Plate slips	1.24 × 10^1^	2.09 × 10^2^
Open plate drop	1.62 × 10^7^	4.09 × 10^9^

^*a*^ Values represent the estimated cumulative inhaled viral dose over a 10-minute exposure duration, calculated using virus concentrations recovered from the gelatin filters and a modeled human inhalation rate of 9.5 L/min. Values are presented as active infectious units (PFU) and genomic equivalents (ePFU). Notably, ePFU values represent the total genomic fragments rather than viable virions. Additional assumptions and limitations are provided in Materials and Methods.

Estimated virus exposures varied by over six orders of magnitude across the tested MS2 actions. The simulated accidental plate drop represented the highest potential for exposure to infectious virus, with an estimated dose of 1.62 × 10^7^ PFU over 10 min. This was nearly four orders of magnitude greater than the dose estimated for routine micropipetting (1.24 × 10^3^ PFU), which yielded the highest infectious virus exposure among the routine laboratory procedures. In contrast, serological pipetting and plate slips resulted in the lowest estimated doses. Notably, total viral particle exposure (ePFU) was consistently 10^1^ to 10^2^ higher than infectious virus exposure (PFU), reflecting the specific total-to-infectious MS2 virus ratio. Given that this ratio between genomic copies and viable virus is pathogen-specific and varies across human viral pathogens, providing both ePFU and PFU values establishes a broader range for characterizing inhalation risks across different viruses.

## DISCUSSION

This study presents the first characterization of viral aerosol generation during laboratory procedures and simulated accidents using a viral surrogate. Prior research on aerosol generation in laboratories has used bacterial surrogates or physical tracers (e.g., culture media), leaving a significant gap in understanding viral aerosol generation risks in laboratories. By integrating a high-resolution particle sizer (APS), a cascade impactor (NGI), and gelatin filters within a controlled testing chamber, we establish a framework for quantifying viral aerosol generation.

A central finding here is that every tested experimental action, whether routine or accidental, generated detectable aerosols across a broad size spectrum. Notably, a persistent submicron peak near 0.6 µm was observed across all NaFl and MS2 experimental actions, indicating a shared aerosolization mechanism. Additionally, multi-modal aerosol size distributions were observed, which can be attributed to several concurrent aerosolization mechanisms. Those mechanisms may include bubble-cap film bursts, cavity collapses, jet fragmentation, and spume drops (23–26). General particle size distribution trends included infectious virus (PFU) aligning with mass-based plots and total virus (ePFU) aligning with number-based plots. In polydisperse distributions, the mass contribution of larger particles can effectively obscure the smaller particle peaks in mass-based plots. For instance, the 0.6 µm peak in the plate drop number concentration plot (Fig. 9A) is not apparent in the mass concentration plot (Fig. 9B), but smaller particles remain distinct in number concentration plots, highlighting the importance of reporting both number and mass concentrations. Since smaller particles generally exhibit lower deposition rates and higher environmental persistence (27), relying solely on mass-based plots may lead to an underestimated long-term inhalation risk assessment.

A consistent pattern emerged where viral RNA concentrations (ePFU) exceeded infectious concentrations (PFU) across all samples. This is likely due to the mechanical stresses inherent in aerosolization (20), such as shear forces and rapid desiccation, which can fragment virions and release exogenous nucleic acids. Despite an apparent reduction in viability, the recovery of infectious MS2 spanning multiple particle sizes during all MS2 actions is a significant biosafety finding.

Every tested MS2 action produced viral aerosols within size fractions capable of depositing in the head airway, tracheobronchial, and alveolar regions (27, 28). When considering routine laboratory pipetting procedures, often completed multiple times within a day, the risk of infectious aerosol generation risks is further underscored by these results: a single 0.1 mL transfer with a micropipette generated 13.4 PFU/L air, and a 2 mL transfer with a serological pipette generated 1.2 PFU/L air. Considering that many high-consequence pathogens possess a low ID_50_, these results provide strong support for the recommendation of using biological safety cabinets (BSC) for all manipulations of infectious materials (29).

The NaFl actions with Avicel-supplemented matrices suggest that liquid viscosity and composition influence aerosolization dynamics. Higher viscosity generally resulted in lower mass concentrations and a shift in aerosol size modes toward larger diameters. This suggests that accidental aerosol generation may change depending on the suspension fluid, which is important since viruses are often suspended in different matrices, such as a saline buffer, culture media, or a clinical specimen. Furthermore, as previous studies have found that matrices influence virus stability in aerosols (20, 30), highlighting the importance of considering matrices when comparing aerosol generation between studies.

Beyond the characterization of suspended aerosols, qualitative observations during the NaFl screening phase demonstrated that high-energy experimental actions generate larger droplets that rapidly fall out of suspension (e.g., plate drops and pipette drops). Although these larger droplets deposited on the chamber base and were not measured at the sampling instruments, they represent a surface contamination hazard in a laboratory. Consequently, these accidents present a dual exposure pathway, encompassing both inhalation risks from the suspended respirable fraction and fomite-mediated transmission risks via the contact route.

While the controlled chamber utilized in this study improves upon previous open-air sampling by providing a controlled environment, the upwelling flow profile creates distinct exposure dynamics compared to an open bench or a BSC. A functional BSC uses targeted airflows to capture and exhaust particulate matter, while an open bench is subject to variable room dilution and settling. This chamber’s upwelling flow was intentionally designed to minimize particle loss and maximize collection efficiency, meaning that the concentrations recovered at the gelatin filter capture the full spectrum of aerosolized virus. Therefore, these values should be interpreted as a conservative, maximum-release scenario rather than an exact replication of exposure. Additionally, the observed virus concentrations are limited to the MS2 surrogate, so other viruses such as SARS-CoV-2 likely exhibit different environmental persistence. To account for these variations and mitigate the bias inherent in surrogate selection, we report both nucleic acid and infectious virus concentrations to provide a range for risk assessments.

Overall, these findings underscore the need for continued vigilance in laboratory biosafety practices and provide a quantitative foundation for developing predictive frameworks to assess aerosol risks in laboratories. As virology research expands globally, particularly concerning emerging and high-consequence pathogens, understanding the physical and biological dynamics of viral aerosol generation is needed to protect research personnel while enabling scientific progress.
